# Alzheimer's Disease Diagnosis With Brain Structural MRI Using Multiview-Slice Attention and 3D Convolution Neural Network

**DOI:** 10.3389/fnagi.2022.871706

**Published:** 2022-04-26

**Authors:** Lin Chen, Hezhe Qiao, Fan Zhu

**Affiliations:** ^1^Chongqing Key Laboratory of Big Data and Intelligent Computing, Chongqing Institute of Green and Intelligent Technology, Chinese Academy of Sciences, Chongqing, China; ^2^University of Chinese Academy of Sciences, Beijing, China

**Keywords:** Alzheimer's disease (AD), disease prognosis, multi-view-slice attention, 3D convolution neural network, brain sMRI image

## Abstract

Numerous artificial intelligence (AI) based approaches have been proposed for automatic Alzheimer's disease (AD) prediction with brain structural magnetic resonance imaging (sMRI). Previous studies extract features from the whole brain or individual slices separately, ignoring the properties of multi-view slices and feature complementarity. For this reason, we present a novel AD diagnosis model based on the multiview-slice attention and 3D convolution neural network (3D-CNN). Specifically, we begin by extracting the local slice-level characteristic in various dimensions using multiple sub-networks. Then we proposed a slice-level attention mechanism to emphasize specific 2D-slices to exclude the redundancy features. After that, a 3D-CNN was employed to capture the global subject-level structural changes. Finally, all these 2D and 3D features were fused to obtain more discriminative representations. We conduct the experiments on 1,451 subjects from ADNI-1 and ADNI-2 datasets. Experimental results showed the superiority of our model over the state-of-the-art approaches regarding dementia classification. Specifically, our model achieves accuracy values of 91.1 and 80.1% on ADNI-1 for AD diagnosis and mild cognitive impairment (MCI) convention prediction, respectively.

## 1. Introduction

Alzheimer's disease (AD) is the most common cause of dementia that causes progressive and permanent memory loss and brain damage. It is critical to initiate treatment for slowing down AD development in early AD. As a non-contact diagnostic method, structural magnetic resonance imaging (sMRI) is regarded as a typical imaging biomarker in quantifying the stage of neurodegeneration (Kincses et al., 2015; Bayram et al., 2018; Shi et al., 2018). Based on the examination of the brain's sMRI images, numerous artificial intelligence (AI) technologies, including conventional voxel-based machine learning methods and deep-learning-based approaches, have been performed for assisting the cognitive diagnosis (Martí-Juan et al., 2020; Tanveer et al., 2020; Wu et al., 2021a,b).

In the early attempts, traditional statistical methods based on voxel-based morphology (VBM) were introduced to measure the brain's morphologic changes. VBM-based studies determine the intrinsic characteristics of specific biomarkers, such as the hippocampus volumes (Fuse et al., 2018), cortex sickness (Luk et al., 2018), subcortical volumes (Vu et al., 2018), and frequency features with non-subsampled contourlets (Feng et al., 2021), to calculate the regional, anatomical volume of the brain. However, most VBM-based approaches relying on domain knowledge and expert's experience need a complex handcrafted feature extraction procedure, which is independent of the subsequent classifiers, resulting in potential diagnostic performance degradation.

With the advancement of deep learning, especially the successful applications of convolution neural networks (CNN), in recent years, a growing body of research employed deep learning to analyze the MR images by training an end-to-end model without handcrafted features (Zhang et al., 2020; AbdulAzeem et al., 2021; Qiao et al., 2021). Since the 3D volumetric nature of sMRI, 3D-CNN could be directly applied to capture the structural changes of the whole brain at the subject-level (Jin et al., 2019). However, there is much useless information in the complete MRI with millions of voxels. Furthermore, it is hard to fully train the CNNs with only a few labeled MRI data available at the subject level. Many deep-learning-based methods turn to exact pre-determination of regions-of-interest (ROI) for training the models with 3D-Patch or 2D-slice (Ebrahimighahnavieh et al., 2020). Liu et al. (2020b) extract multi-scale image patches based on the pre-determined anatomical landmarks from sMRI for training an end-to-end CNN. Lian et al. (2020a,b) trained multiple classifiers with multilevel discriminative sMRI features from the whole sMRI with a hybrid network to capture local-to-global structural information. Compared with the modeling in the subject level, the patches or slices carry more local features but lose some global information. In addition, some studies try to exclude irrelevant regions by emphasizing specific brain tissues with the help of segmentation technology. Cui and Liu (2019) and Poloni and Ferrari (2022) focus on the specific biomarker from specific regions, such as the hippocampus, to capture the structural changes in 3D MR images for AD and mild cognitive impairment (MCI) classification. Chen and Xia (2021) design a sparse regression module to identify the critical cortical regions, such as the amygdala, posterior temporal lobe, and propose a deep feature extraction module to integrate the features landmarked regions for the diagnosis process. However, such methods need extra tissue segmentation operations, which inevitably increase the complexity of the diagnostic model.

**Figure 1:**
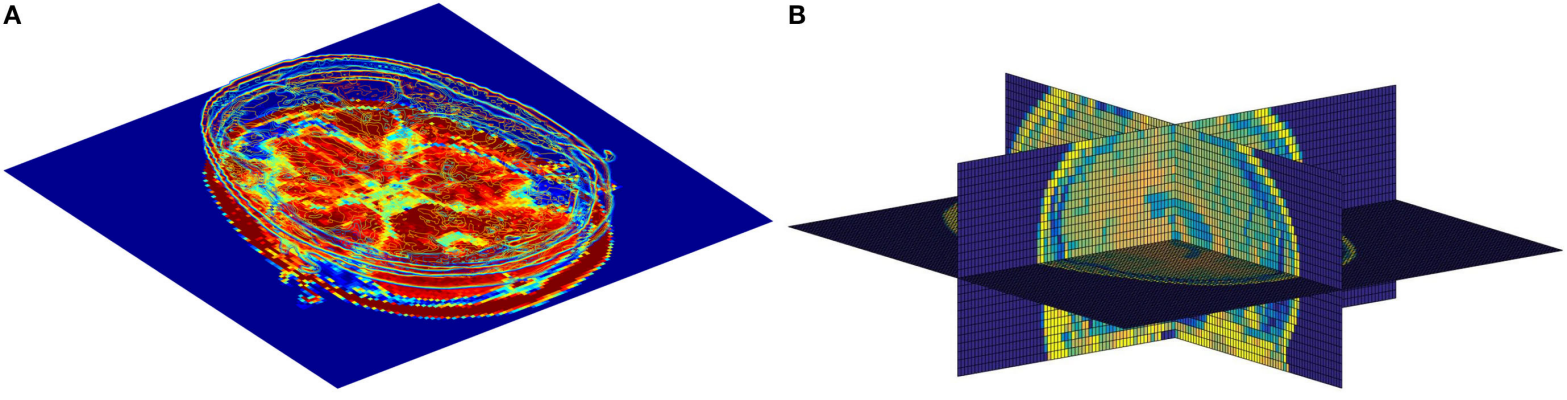
The slice-level information in brain sMRI. **(A)** Slice-level features in axial plan. **(B)** Slice-level features captured in multiview, including the sagittal, coronal, and axial planes.

Although the existing models have achieved outstanding results so far, it is still a challenging work for AD diagnosis due to a large number of volumes in 3D MR images and a subtle difference between abnormalities and normality brains, i.e., it is vital to extract subtle changes in disease progression from MRI sequence data with a high denominational. Previous studies focus on extracting features from the whole brain or individual slices separately, ignoring the feature complementarity from different views. As illustrated in Figure 1, each slice of the brain sMRI in different views contains a certain amount of local information that could also be valuable for dementia diagnosis. Considering both global structure changes of whole brain and fine-grained local distinctions of slices could be both crucial, this study proposes a novel fusion model for AD classification, named multiView-slice attention and 3D convolution neural network (MSA3D), which organically integrates multiple slices features and 3D structural information.

The main contributions of this study are three-fold:

1. We proposed an MSA3D model to combine the 2D multi-view-slice levels and global 3D subject-level features for fully mining the subtle changes in different views and dimensions.
2. We propose a slice-level attention module to help the CNN focus on specific slices to obtain more discriminative features representations from abundant vowels.
3. We perform two classification tasks, i.e., AD diagnosis and MCI conversion prediction, on two ADNI datasets. Our model achieves superior diagnostic results compared with other tested models, demonstrating our model's efficacy in aiding dementia prediction.

## 2. Materials and Data Preprocessing

### 2.1. Studied Subjects

Following the previous studies (Liu et al., 2019; Lian et al., 2020b), we employed two public sMRI data sets, i.e., ADNI-1 and ADNI-2, for empirical study. Both of them can be found on the Alzheimer's Disease Neuroimaging Initiative (ADNI) website (Jack et al., 2008). This study employed the ADNI data only for model validation but did not involve any patient interaction or data acquisition. More detailed data acquisition protocols are available at http://adni.loni.usc.edu/. We collected a total of 1,451 subjects from the ADNI database with baseline T1 weighted (T1W) brain MRI scans, which are divided into four categories:

- Cognitively Normal (CN): Subjects diagnosed with CN at baseline and showed no cognitive decline.
- Stable MCI (sMCI): Subjects diagnosed with MCI remain stable and have not converted to AD at all time-points (0–90 months).
- Progressive MCI (pMCI): Subjects are diagnosed with MCI who would gradually progress to AD within 0–36 months.
- Alzheimer's disease: Subjects diagnosed as AD at baseline and whose conditions would not change during the follow-up period.

To avoid data leakage problems mentioned in Wen et al. (2020), we also remove the subjects exited in both ADNI-1 and ADNI-2. More specifically, the ADNI-1 dataset is formed of 808 subjects with 1.5 T T1W sMR brain images, including 183 AD, 229 CN, 167 pMCI, and 229 sMCI. The ADNI-2 dataset has 643 3T T1W sMR brain images, including 143 AD, 184 CN, 75 pMCI, and 241 sMCI. Table 1 summarizes the detailed clinical information of the studied subjects, including age, sex, and the scores of the mini-mental state examination (MMSE). In our experiments, these two independent datasets will be employed as the training dataset and testing dataset, repetitively, to perform cross-validation. More specifically, we first trained the model on the ADNI-1 and evaluated it on ADNI-2. Subsequently, we reversed the experimentation and used the ADNI-2 for model learning, and then the trained model was assessed on ADNI-1. Note that we employed the ADNI data only for empirical analysis but this study did not employ any patient interaction or data acquisition.

**Table 1:** Detailed clinical information of the studied subjects in ADNI-1 and ADNI-2 (± means the SD).

**Dataset**	**Label**	**Total number**	**Age (Years)**	**Sex (M/F)**	**MMSE**
ADNI-1	NC	229	76.2 ± 5.1	119/110	29.2 ± 1.0
	sMCI	229	74.8 ± 7.6	153/76	27.2 ± 1.7
	pMCI	167	74.9 ± 7.2	102/65	26.9 ± 1.7
	AD	183	75.6 ± 7.6	96/87	23.1 ± 2.5
ADNI-2	NC	184	77.3 ± 6.7	87/97	28.8 ± 1.7
	sMCI	241	71.3 ± 7.5	134/107	28.3 ± 1.5
	pMCI	75	71.9 ± 7.2	40/35	27.0 ± 1.6
	AD	143	75.6 ± 7.8	85/58	21.9 ± 3.8

### 2.2. Data Preprocessing

The standard preprocessing pipeline was performed on all the T1W brain MRIs as follows: First, all MRIs were performed in an axial orientation parallel to the line through anterior commissure (AC)-posterior commissure (PC) correction. Then the invalid volumes of the sMRI, i.e., the blank regions, were removed, leaving only the brain tissues. Subsequently, the intensity of brain images was corrected and normalized with the N3 algorithm after the skull dissection (Wang et al., 2011). Finally, all the aligned images are resized into the same spatial resolution for facilitating the CNN training. The model's inputs are fixed to 91 × 101 × 91(i.e., 2*mm* × 2*mm* × 2*mm* cubic size) in our experiment, following the previous study (Jin et al., 2020).

## 3. Methodology

**Figure 2:**
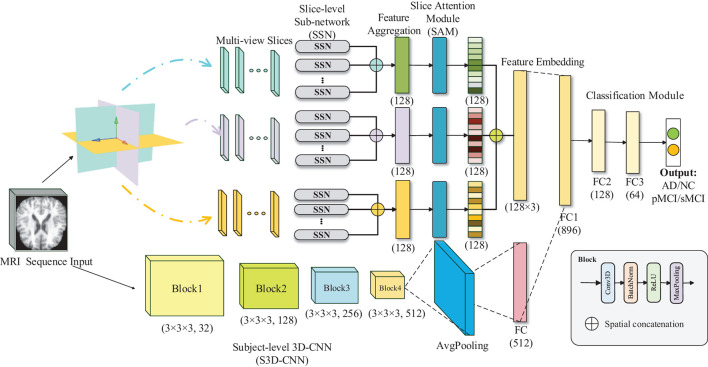
Illustration of the proposed multiview-slice attention and 3D convolution neural network (MSA3D) model.

The overall architecture of our model is presented in Figure 2, which is composed of five main parts: the MRI sequences input, multi-view-slice sub-network (MVSSN), slices attention module (SAM), subject-level 3D-CNN (S3D-CNN), and a softmax classifier with full connection layer. The following sections provide more details for each module.

### 3.1. Multi-View-Slice 2D Sub-Networks

**Figure 3:**
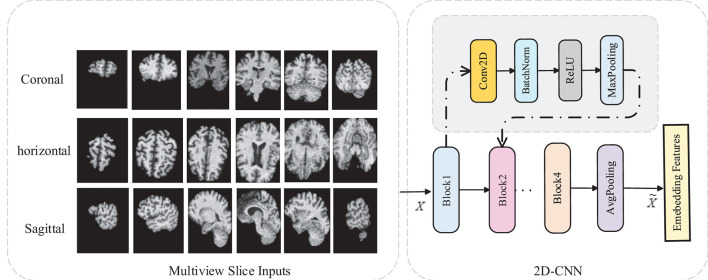
2D-CNN slice sub-network for multi-view slice features extraction.

In this subsection, we introduce the MVSSN module for extracting multiview 2D-slice level features. As shown in Figure 3, the inputs of MVSSN are consist of the MR slices in three views, i.e., the sagittal, coronal, and axial imaging planes. Since discriminative features may exist in different slices, we employ a 2D-CNN to extract the multiview slice features from each slice. Let's denote the *x*, *y*, and *z* as the MRI planes of sagittal, coronal, and axial, respectively, particularly, ${\text{S}}_{x}\;{=[\text{s}}_{x}^{1}{\text{, s}}_{x}^{2},\ldots ,s_{x}^{M_{x}}]$ denotes the slice cluster in the *x* plane, where *M*_*x*_ is the total slice number of the cluster **S**_*x*_. After using the multiple 2D-CNNs on each slice to generate the feature maps in different views separately, the input *I* ∈ *R*^*D* × *H* × *W*^ can be transformed as the feature maps *F*_*x*_, *F*_*y*_, *F*_*z*_ in three dimensions. For example, each feature map $F_{x}^{i}$ in sagittal view is calculated by Equation (1):

(1) $$\begin{array}{l} \begin{array}{l} F_{x}^{i}=f_{x}^{i}\left(s_{x}^{i},w_{x}^{i}\right) \end{array} \end{array}$$

where $f_{x}^{i}$ is a independent 2D-based CNN, $w_{x}^{i}$ is the weight of CNN $f_{x}^{i}$, and *i* ∈ [1, *M*_*x*_] means the *i*th slice in the *x*-direction. Each $f_{x}^{i}$ contains three CNN blocks, each with a conventional layer, a barch normalize (BN) layer, a rectified linear unit (RELU) operator, and a maxpooling layer. Detailed parameters of our 2D-based CNN are listed in Table 2.

**Table 2:** Detailed parameters of our 2D-CNN slice sub-network.

**Layer**	**Kernel**	**Stride**	**Activation**	**Output channels**
Conv2D	3 × 3	2	BachNorm+Relu	8
MaxPooling2D	2 × 2			8
Conv2d	3 × 3	2	BachNorm+Relu	32
MaxPooling2D	2 × 2			32
Conv2D	3 × 3	2	BachNorm+Relu	64
MaxPooling2D	2 × 2			64
Global-Avg-Pooling2D	1 × 1	1		128
Full connected				128
Full connected				8

After the Global-Avg-Pooling (GAP) operation, the feature map $F_{x}^{i}$ can be pooled as a vector denoted as $I_{x}^{i}$. In the end, all the feature maps in *x* view can be cascaded as ${\text{I}}^{x}=\left[I_{1}^{x},I_{2}^{x},\ldots ,I_{M_{x}}^{x}\right]$. The same conventional operation can be applied on *y* and *z* views to generate the corresponding feature map clusters.

### 3.2. Slices Attention Module

Each vector in **I**^*k*^ can be regarded as a class-specific response after extracting the multiple slices-level features using the MVSSN. Considering that the volumetric MRI data contains different slices, many of them may not contain the most representative information relevant to dementia (Lian et al., 2021). To address this issue, we proposed a SAM to help the CNN focus on the specific features by exploiting the interdependencies among slices.

As shown in Figure 2, given a set of features embedding of the *j*^*th*^ direction, denoted as ${\text{I}}^{k}\in^{M_{k}\times C}$, where C = 8 is the feature channels of each slice, and *k* ∈ {*x, y, z*} means the MR plane. By employing an attention mechanism, we can obtain the slice attention ${\text{A}}^{k}\in^{M_{k}\times M_{k}}$, which can build the dynamic correlations between the target diagnosis label and slice-level features with the following equation:

(2) $$\begin{array}{l} a_{ij}^{k}=\frac{\exp\left(I_{i}^{k}\cdot I_{j}^{k}\right)}{\sum_{i=1}^{M_{k}}\exp\left(I_{i}^{k}\cdot I_{j}^{k}\right)} \end{array}$$

where $a_{ij}^{k}\in {\text{A}}^{k}$ is the score that semantically represents the impact of *i*^*th*^ slice feature on the *j*^*th*^ slice in the *k*^*th*^ direction. The final output of the weighted slice features ${\tilde{\text{I}}}^{k}\in^{M_{k}\times C}$ can be calculated by:

(3) $$\begin{array}{l} {\tilde{I}}_{j}^{k}=\beta \overset{M_{k}}{\underset{i=1}{\sum }}\left(a_{ij}^{k}I_{i}^{k}\right)+I_{j}^{k} \end{array}$$

where β is a learnable parameter that will gradually increase from 0, note that the final output feature maps are the sum of all the weighted features of the slices in one direction so that the SAM can adaptively emphasize the most relevant slices to produce a better AD inference.

After the SAM module, we fuse all the slice features in three directions using concatenation operation to form the final slice-level features ${\text{F}}_{\text{s}}=\left[{\tilde{\text{I}}}^{x},{\tilde{\text{I}}}^{y},{\tilde{\text{I}}}^{z}\right]$, where **F**_**s**_ represents the cascaded weighted features which can capture the multiple views of local changes of the brain in three directions in 2D MRI images.

### 3.3. Subject-Level 3D Neural Network

The brain MRI data can be regarded as 3D data with an input size of *H* × *W* × *D*, where *H* and *W* denote the height and width of the MRI, repetitively, and *D* is the image sequence. In order to explore the global structure changes of the brain, all of the convolution operations and pooling layers are reformed from 2D to 3D. The 3D CNN operator is given in Equation (4):

(4) $$\begin{array}{l} \begin{array}{l} u_{j}^{l}\left(x,y,z\right)=\\ \underset{\delta_{x}}{\sum }\underset{\delta_{y}}{\sum }\underset{\delta_{z}}{\sum }F_{i}^{l-1}\left(x+\delta_{x},y+\delta_{y},z+\delta_{z}\right)\times W_{ij}^{l}\left(\delta_{x},\delta_{y},\delta_{z}\right) \end{array} \end{array}$$

where (*x, y, z*) refers to the 3D coordinates in sMRI data, $F_{i}^{l-1}$ is the *i*th feature map of the *l* − 1 layer. $W_{ij}^{l}\left(\delta_{x},\delta_{y},\delta_{z}\right)$ is a 3D convolution kernel slides in 3 dimensions, thus the new *j*th feature map $u_{j}^{l}\left(x,y,z\right)$ of the *l* layer can be generated after 3D convolution across the $F_{i}^{l-1}$ from the *l* − 1 layer. Similar to the 2D-CNN, our 3D-CNN includes four network blocks, and each block has a 3D-CNN layer, 3D BN layer, ReLu activation, and 3D max-pooling layer. Finally, the 3D convolutional feature maps are pooled into one 1D vector using a 3D-GAP layer with a kernel size of 1 × 1 × 1. The produced vector represents the global subject-level features. Detailed parameters of our 3D-CNN subject-level subnetwork are shown in Table 3.

**Table 3:** Detailed parameters of our 3D-CNN subject sub-network.

**Layer name**	**Kernel**	**Stride**	**Activation**	**Output channels**
Conv3D	3 × 3 × 3	1	BachNorm3d+Relu	32
MaxPooling3D	3 × 3 × 3	2		32
Conv3D	3 × 3 × 3	1	BachNorm3d+Relu	128
MaxPooling3D	3 × 3 × 3	2		128
Conv3D	3 × 3 × 3	1	BachNorm3d+Relu	256
MaxPooling3D	3 × 3 × 3	2		256
Conv3D	2 × 2 × 2	2	BachNorm3d+Relu	512
MaxPooling3D	5 × 5 × 5	2		512
Globel-Avg-Pooling3D	1 × 1 × 1			512

### 3.4. Fully Connected Layer and Loss for Classification

To exploit both the slice-level and subject-level features generated by 2D and 3D-CNNs, a fully connected (FC) layer is employed to concatenate all the 2D and 3D features maps, followed by a final FC layer and a softmax classifier, which outputs the prediction probability of the diagnostic labels. The cross-entropy (CE) is widely adopted as the training loss function for image classification (Liu et al., 2021), which is given as follows:

(5) $$\begin{array}{l} L=-\frac{1}{C}\overset{C}{\underset{c=1}{\sum }}\frac{1}{N}\underset{X_{i}\in \text{X}}{\sum }\text{I}\left\{Y_{i}^{c}=c\right\}\log\left(\text{P(}Y_{i}^{c}=c|X_{i}:\text{W})\right) \end{array}$$

where I{·} = 1 if {·} is true, otherwise I{·} = 0. *N* is the total number of test subjects and *X*_*i*_ means the *i*th sample with the corresponding label *Y*_*i*_ in the training datasets **X**, and *i* ∈ [1, *N*]. $\text{P(}Y_{i}^{c}=c|X_{i}:\text{W})$ measures the probability of the input sample *X*_*i*_ that is correctly classified as the $Y_{i}^{c}$ by the trained network with weights **W**.

### 3.5. Complexity Analysis

We further analyze our proposed model's complexity by reporting the two branches of subnetworks, respectively. For the aspect of the global subject-level 3D-CNN model, the computational complexity of 3D-CNN layer is $O\left(D_{x}D_{y}D_{z}K_{global}^{3}\right)$, where *K*_*global*_ is 3D-CNN kernel size, while *D*_*x*_, *D*_*y*_, *D*_*z*_ is the feature map dimensions of the layer. For the aspect of the slice-level 2D-CNN model, since the 2D feature maps are fused in three dimensions, the time complexity of the 2D-CNN layer is $O\left(M_{z}D_{x}D_{y}K_{slice}^{2}+M_{x}D_{y}D_{z}K_{slice}^{2}+M_{y}D_{x}D_{z}K_{slice}^{2}\right)$, where *M*_*x*_, *M*_*y*_, *M*_*z*_ denotes the total number of slices in three MR planes, receptively, and *K*_*slice*_ is the 2D-CNN kernel size.

## 4. Experimental Results

### 4.1. Competing Methods

We first compare our proposed MSA3D method with multiple deep-learning-based diagnosis approaches that we reproduced and evaluated on the same training and testing datasets including (1) a statistical method based on VBM with SVM [denoted as VBM+SVM, proposed by Ashburner and Friston (2000)], (2) a method using 3D-CNN features [denoted as 3D-CNN, proposed by Wen et al. (2020)], (3) a method using multi-slice 2D features, i.e., the features extracted from all the slices in three directions (denoted as Multi-Slice), and (4) a method using 3D-CNN with 3D patch-level features (denoted as Multi-Patch).

1. Voxel+SVM: As a conventional statistical-based model, Voxel+SVM performed sMRI analyses at the voxel level (Ashburner and Friston, 2000). Using a non-linear image registration approach, we first normalized all MRIs with the automated anatomical atlas (AAL) template. Then, we segmented the gray matter (GM) from sMRI data. In the end, we mapped the density of GM tissue into one vector and used the support vector machine (SVM) as the classifier for AD diagnosis.
2. 3D convolution neural network: As an important part of MSA3D, 3D-CNN can extract global subject-level changes of sMRI for dementia diagnosis (Wen et al., 2020). Thus, it can be regarded as the baseline model in our study. In this model, we only give the 3D MRI data as the input for training the 3D-CNN.
3. Multi-Slice: As another essential component of MSA3D, the multi-slice model focus on the local slice-level features, which consist of all the features extracted by using the 2D-CNN with the 2D slices in sagittal, coronal, and axial MR planes.
4. Multi-Patch: In this method, multiple 3D-patches are partitioned from the whole brain according to the landmarks defined in Zhang et al. (2016) and Liu et al. (2020b) to extract region-scale features (ROI), and then we train a 3D-CNN as the feature extractor for each patch. In the end, all the ROI-based features were cascaded to obtain the final embedded feature for the entire sMRI.

### 4.2. Experimental Setting

All the tested models are implemented with Python on Pytorch using one NVIDIA GTX1080TI-11G GPU. During the training stage, the batch size is set to the same value of 12 for all models for a fair comparison. Stochastic gradient descent (SGD) with an initial learning rate of 0.01 and a weighted delay of 0.02 is adopted as the optimization approach, along with an early stopping mechanism for avoiding over-fitting. The following five criteria are calculated to investigate the performance of the tested models, including accuracy (ACC), specificity (SPE), sensitivity (SEN), the area under the ROC curve (AUC), and F1-values (F1).

### 4.3. Results on ADNI-2

**Figure 4:**
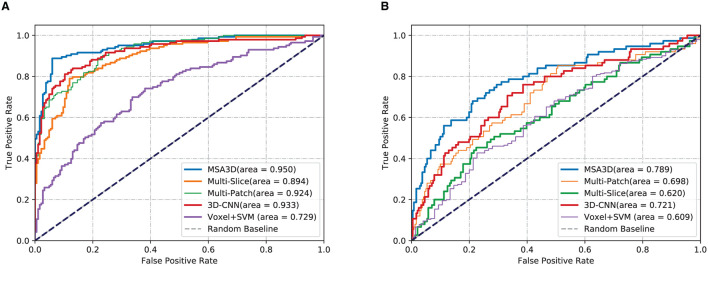
Comparisons results in terms of ROC curves. The models are trained on ADNI-1 and tested on ADNI-2. **(A)** AD vs. NC. **(B)** pMCI vs. sMCI.

We first present the comparison results of two classification tasks (i.e., AD vs. NC and pMCI vs. sMCI) on ADNI-2 in Table 4 and Figure 4, with the tested methods trained on the ADNI-1. As we can inform from Table 4, Multi-Patch shows a better performance than Multi-Slice on AD prediction, especially on the challenging pMCI vs. sMCI. The results indicate that local discriminative features are important for MCI prediction, and only the 2D-slice level features may not be a good option for CNNs. In addition, 3D-CNN achieved the second-best results on both AD and MCI prediction tasks. We can also find that all the deep-learning-based models perform better than the conventional Voxel+svm method. The main reason is that the deep-learning-based technique can achieve a better feature extraction with an end-to-end framework. In general, our model consistently yields better performance than the tested methods, e.g., in the case of MSA3D vs. 3D-CNN baseline, our model resulted in 7 and 5.6% improvements in terms of ACC and AUC for classifying AD/NC, and 7.3% and 16.9% improvements in terms of ACC and AUC for determining pMCI/sMCI. This result shows that after fusion of the 2D and 3D information through two branches of CNNs, our model can capture more discriminative changes in both multiview 2D-slices and 3D whole-brain volumes in the progress of AD and MCI conversion. So that our model generates significant improvements in terms of all the metrics compared to other methods in comparison.

**Table 4:** Classification results of AD vs. CN and MCI convention on ADNI-2.

**Method**	**AD vs. CN**					**pMCI vs. sMCI**				
	**ACC**	**SEN**	**SPE**	**AUC**	**F1**	**ACC**	**SEN**	**SPE**	**AUC**	**F1**
Voxel+SVM	0.759	0.677	0.810	0.729	0.705	0.736	0.107	0.769	0.609	0.162
3D-CNN	0.872	0.874	0.839	0.933	0.856	0.769	0.427	0.831	0.721	0.467
Multi-Slice	0.838	0.755	0.826	0.894	0.813	0.728	0.267	0.792	0.620	0.317
Multi-Patch	0.841	0.790	0.844	0.924	0.803	0.722	0.373	0.821	0.698	0.438
MSA3D	**0.911**	**0.888**	**0.914**	**0.950**	**0.898**	**0.801**	**0.520**	**0.856**	**0.789**	**0.553**

*All the models are trained on ADNI-1. The best results are highlighted in bold*.

### 4.4. Results on ADNI-1

**Figure 5:**
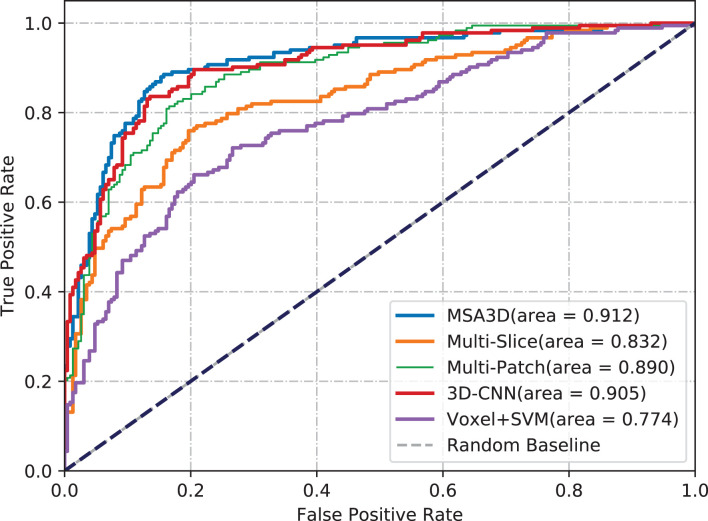
Comparisons of ROC curves. The models are trained on ADNI-2 and tested on ADNI-1.

In order to further investigate the effectiveness of the test models, we also perform a cross-valuation on ADNI datasets, i.e., we trained the models on ADNI-2 and tested them on ADNI-1. It needs to be pointed out that because of the lack of sufficient pMCI samples in ADNI-2 (75 in ADNI-2 vs. 167 in ADNI-1), we only conduct the experiments of AD diagnosis on ADNI-1. The comparison results are summarized in Table 5 and Figure 5, from which we can observe similar results compared to the models tested on the ADNI-2. Our model still produces the best values in terms of all the metrics compared with the other methods.

**Table 5:** Classification results of AD vs. CN on ADNI-1.

**Methods**	**ACC**	**SEN**	**SPE**	**AUC**	**F1**
Voxel+SVM	0.754	0.728	0.781	0.774	0.741
3D-CNN	0.833	0.738	0.813	0.905	0.796
Multi-slice	0.774	0.776	0.812	0.832	0.753
Multi-patch	0.808	0.710	0.793	0.890	0.767
MSA3D	**0.864**	**0.858**	**0.884**	**0.912**	**0.849**

*All the models are trained on ADNI-2. The best results are highlighted in bold*.

Meanwhile, we can find a significant performance drop for all models when trained on ADNI-2, which leads to a relatively small improvement of AUC achieved by our model compared with the 3D-CNN. The main reason for this is that ADNI-1 and ADNI-2 were collected using 1.5 and 3.0 Tesla MRI scanners, respectively. The strength of a 3.0 T magnet is two times that of a 1.5 T magnet, which could cause the overestimation of brain parenchymal volume at 1.5 T (Chu et al., 2016). The variable image quality between different scanners directly impacts the models for diagnosis. However, our model still outperforms the 3D-CNN baseline by 5.3% of the F1 value in this scenario. All of these findings suggest the proposed model's efficacy and reliability.

### 4.5. Comparison With Other Methods in Literature

In this section, we give a brief description of our MSA3D method with the previous study reported in the literature for AD diagnosis using the ADNI database. The state-of-the-art comparison studies contain:

1. The conventional statistical-based methods include: SVM trained with Voxel-based features (VBF; Salvatore et al., 2015); landmark-based morphometric features extracted from a local patch (LBM; Zhang et al., 2016); SVM trained with landmark-based features (SVM-landmark; Zhang et al., 2017).
2. The deep-learning-based methods include: 3D-CNN based on the whole brain sMRI data (whole-3DCNN; Korolev et al., 2017); Multi-layer perception + recurrent neural network using the longitudinal sMRI features (MLP-RNN; Cui et al., 2018); 3D-CNN based on the multiple-modality inputs including sMRI, PET, and MD-DTI data (multi-3DCNN; Khvostikov et al., 2018); 3D-DenseNet based on the 3D-patches features extraction from the hippocampal areas (3D-DenseNet; Liu et al., 2020a); hierarchical fully convolutional network based on 3D-patch and regions features extracted with prior landmarks (wH-FCN; Lian et al., 2020b).

As shown in Table 6, We can draw the following conclusions: (1) deep-learning-based methods, especially the CNN-based models, perform much better than most of the conventional statistical methods in terms of ACC. The main reason is that CNN has more feature representation power than handcrafted features. (2) The local features, including ROI-based, landmark-based, and hippocampal segmentation, are also essential to improve the performance of dementia prediction, which indicates that the local changes in whole-brain images provide some valuable clues for AD diagnosis. However, most of these models need predefined landmarks or segmentation regions, which could be hard to obtain potentially informative ROIs due to the local differences between subjects. (3) Different from existing deep-learning-based models (Korolev et al., 2017; Khvostikov et al., 2018; Lian et al., 2020b; Liu et al., 2020a), our proposed model can extract more discriminative features from both local 2D-slice level and 3D-subject level sMRI data using 2D-slice attention network and 3D-CNN, it generates the best ACC, SEN values on AD vs. CN task, and the best SPE and AUC values for predicting pMCI vs. sMCI.

**Table 6:** The performance comparison of our model with other state-of-the-art studies report in the literature using the ADNI database for prediction of AD vs. CN and pMCI vs. sMCI.

**Method**	**Test subjects**	**AD vs. CN**				**pMCI vs. sMCI**			
		**ACC**	**SEN**	**SPE**	**AUC**	**ACC**	**SEN**	**SPE**	**AUC**
VBF	137AD+76sMCI+ 134pMCI+162CN	0.760	–	–	–	0.660			
SVM-Landmark	154 AD+346 MCI +207 CN	0.822	0.774	0.861	0.881	–	–	–	–
LBM	385AD+465sMCI+ 205pMCI+429CN	0.822	0.774	0.861	0.881	0.686	0.395	0.732	0.636
MLP-RNN	198AD+229CN	0.897	0.868	0.925	0.921				
Whole-3DCNN	50AD+77sMCI+ 43pMCI+61CN	0.800	–	–	0.870	0.520	–	–	0.520
Multi-3DCNN	48AD+58CN	0.850	0.880	0.900	–	–	–	–	–
3D-DenseNet	97AD+233MCI +119CN	0.889	0.866	0.808	0.925				
wH-FCN	385AD+465sMCI +205pMCI+429CN	0.903	0.824	0.965	**0.951**	**0.809**	0.526	**0.854**	0.781
Our model	326AD++470sMCI +242pMCI+413CN	**0.911**	**0.888**	0.914	**0.950**	0.801	0.520	**0.856**	**0.789**

*The best results are highlighted in bold*.

It is noteworthy that our model does not need any predefined landmarks or extra location modules (e.g., hippocampus segmentation), but it achieved better or at least comparative diagnostic results than that of existing deep-learning-based AD diagnosis methods. For example, compared with the second-best wH-FCN model, which extracts features from multiple 3D-patches with hierarchical landmarks proposals, MAS3D generates better results in terms of ACC, SEN, and yields almost the same AUC values on AD vs. CN task. For the aspect of the pMCI vs. sMCI task, our model performs slightly worse than the wH-FCN in terms of ACC and SEN. The possible reason is that wH-FCN adopts more prior knowledge to improve the model's recognition capability, i.e., wH-FCN constrains the distances between landmarks and initializes the network parameters of the MCI prediction model from the task of AD classification.

## 5. Discussion

### 5.1. Influence of Features in Different Dimensions

**Figure 6:**
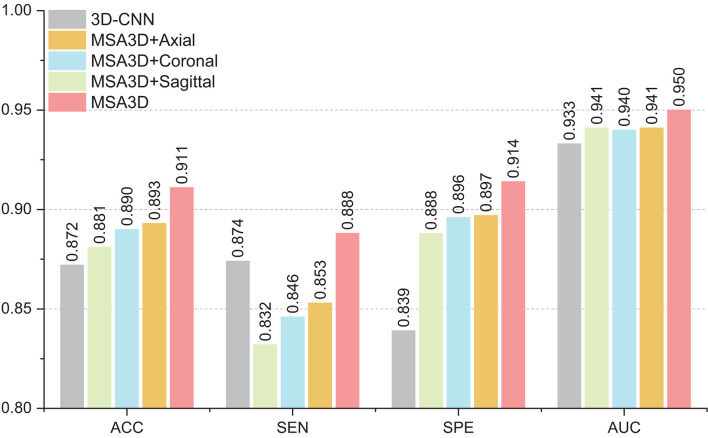
The influence of features fusion in different dimensions.

In this section, we investigate the effects of models using multiple slice-level features in different views for AD classification. As shown in Figure 6, compared with the model combined with features in the axial plane generates much better results than that of the sagittal and the coronal planes in terms of ACC and SEN. Moreover, after combining the features in three dimensions, our proposed MAS3D outperforms all the tested models, especially yielding significantly better SEN values than the tested methods. This result demonstrates that our 2D- and 3D-features fusion strategy can organically integrate the multi-view-slices features in all directions.

### 5.2. Influence of Slice Attention Module

As introduced in Section 3.2, the SAM was employed in our MSA3D model to assist the slice-level feature extraction by exploiting the relationships among the slices, i.e., to filter out uninformative slices efficiently. In this subsection, we conducted an ablation experiment for comparison, in which the SAM is removed from our MSA3D, defined as MS3D, to investigate the effectiveness of the proposed SAM, and all the models are trained using ADNI-1 and obtained the test results on ADNI-2.

**Figure 7:**
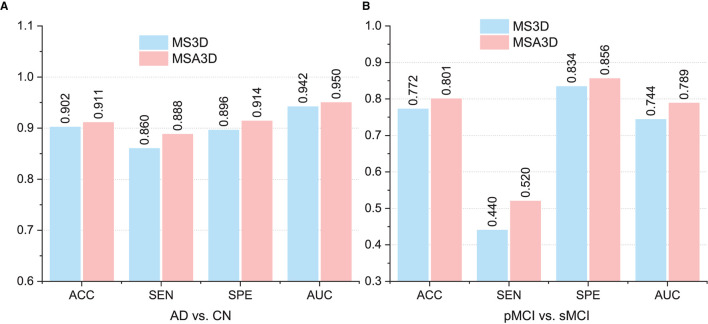
Comparison between multi-view slice fusion without SAM (i.e., MS3D) and multi-view slice fusion with SAM (i.e., MSA3D). **(A,B)** Show the classification results for AD vs. CN and pMCI vs. sMCI, respectively.

The comparison results are illustrated in Figure 7, from which we can inform that: (1) the two variants of our methods (i.e., MS3D and MSA3D) consistently perform better than the baseline model (i.e., 3D-CNN), which means the fusion of 2D -slice level and 3D subject features provides richer feature representation power for AD diagnosis. (2) the SAM further improved the performance of slice level feature extraction, especially on the challenging MCI prediction task, e.g., The proposed MSA3D generally had better classification performances than MS3D (the ACC and SEN is 0.772 vs. 0.801 and 0.440 vs. 0.520, respectively). This indicates that the proposed SAM can help the neural network focus on specific slices and learn more discriminative 2D-slice level features from abundant slices.

### 5.3. Visualization of Slices Features

**Figure 8:**
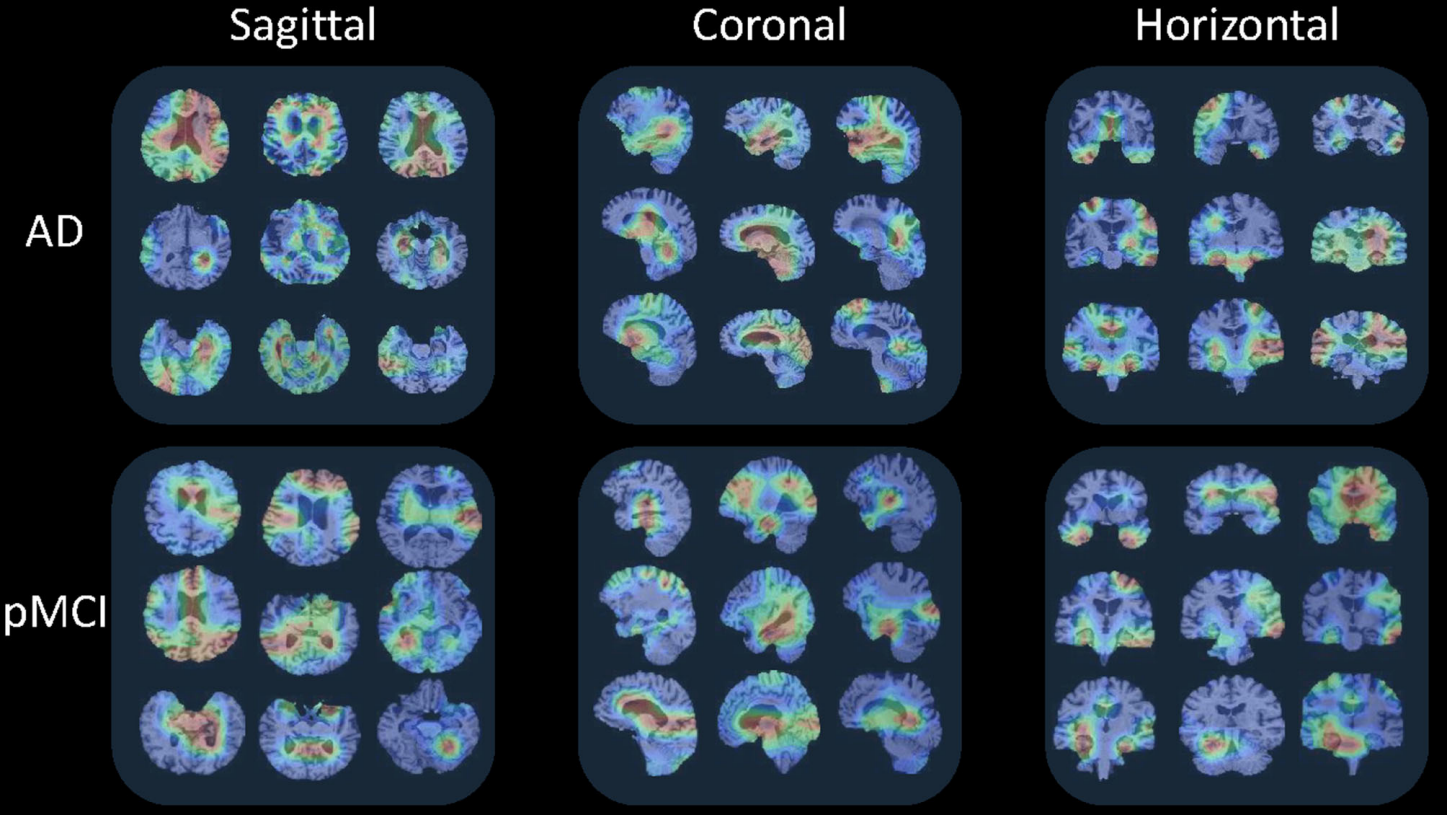
Attention maps of our MAS3D method for predicting multiple subjects selected from the ADNI database with different stages of dementia (i.e., AD and pMCI), respectively. Each subject's attention map is displayed in three MR planes (i.e., sagittal, coronal, and horizontal), where red and blue colors denote high and low discriminative features in sMRI, respectively.

This section visualizes the attention maps produced by our MAS3D method using the Grad-cam (Selvaraju et al., 2020) technology for predicting the subjects with AD and pMCI. The first, second, and third columns of Figure 8 show the different 2D-slices of sMRI in different views, including sagittal, coronal, and horizontal, respectively, where the corresponding model is trained on ADNI-1, and three AD and three pMCI subjects are randomly selected from ADNI-2 for testing.

From Figure 8, we can infer that our model can identify discriminative atrophy areas for different subjects with different stages of dementia, especially for the regions that affect human memory and decision making in the brain. For example, our model emphasizes the atrophy of the frontoparietal cortex, ventricle regions, and hippocampus in the brain. It needs to be pointed out that these highlighted brain regions located by our model in AD diagnosis are consistent with previous clinical research (Chan et al., 2002; Zhang et al., 2021), which have reported the potential sensitive markers for neurodegeneration. All of these results suggest our proposed model can more precisely learn more discriminative features from the brain sMRI for precise dementia diagnosis.

### 5.4. Limitation and Future Study

While the experimental results suggested our proposed model performed well in automatic dementia detection, its performance and generalization might be potentially enhanced in the future by addressing the limitations listed below.

First, we take advantage of both 2D-slice and 3D-subject features in an integrated MSA3D model. However, the numerous 2D slices observably increased the computational complexity. Since not all the slices help determine the prediction, we could reduce the complexity by using an online feature selection module (Wu D. et al., 2021) to select the 2D slices dynamically. Second, the difference distributions between ADNI-1 and ADNI-2 were not taken into account, i.e., 1.5 T scanners and 3 T scanners for ADNI-1 and ADNI-2, repetitively, which might have a detrimental impact on the model's performance, i.e., the model trained on ADNI-2 and assessed on ADNI-1 performed worse than that trained on ADNI-1 and evaluated on ADNI-2. We could potentially introduce the domain adaption technique into our model to reduce the domain gap between different ADNI datasets. Finally, To further verify the generalization capacity of the proposed model, we will investigate more deep-learning-based methods and test our model on other AD datasets for more AD-related prediction tasks, such as dementia status estimation.

## 6. Conclusion

This study explores a 2D-slice-level and 3D subject-level fusion model for AI-based AD diagnosis using brain sMRI. In addition, a slice attention module is proposed to select the most discriminative slice-level features adaptively from the brain sMRI data. The effectiveness of our model is validated on ADNI-1 and ADNI-2, repetitively, for dementia classification. Specifically, our model achieves 91.1 and 80.1% ACC values on ADNI-1 in AD diagnosis and MCI convention precondition, respectively.

## Data Availability Statement

The original contributions presented in the study are included in the article/supplementary material, further inquiries can be directed to the corresponding author.

## Ethics Statement

Ethical review and approval were not required for the study on human participants because all the data in this study were downloaded from the Alzheimer's Disease Neuroimaging Initiative (ADNI) database.

## Author Contributions

LC and HQ implemented and optimized the methods and wrote the manuscript. LC and FZ designed the experiment and algorithm. All authors contributed to the article and approved the submitted version.

## Funding

Publication costs are funded by the National Nature Science Foundation of China under grants (61902370 and 61802360) and are also by the key cooperation project of the Chongqing Municipal Education Commission (HZ2021008 and HZ2021017).

## Conflict of Interest

The authors declare that the research was conducted in the absence of any commercial or financial relationships that could be construed as a potential conflict of interest.

## Publisher's Note

All claims expressed in this article are solely those of the authors and do not necessarily represent those of their affiliated organizations, or those of the publisher, the editors and the reviewers. Any product that may be evaluated in this article, or claim that may be made by its manufacturer, is not guaranteed or endorsed by the publisher.
